# Neonatal Mortality and Education Related Inequality in Cesarean Births in Sub-Saharan Africa: Multi-Country Propensity Score Matching and Meta-Analysis

**DOI:** 10.3390/children9081260

**Published:** 2022-08-20

**Authors:** Tawa O. Olukade, Olalekan A. Uthman

**Affiliations:** 1 Department of Pediatrics, Hamad Medical Corporation, Doha 3050, Qatar; 2 Warwick-Centre for Global Health (WCGH), Division of Health Sciences, Warwick Medical School, University of Warwick, Coventry CV4 7AL, UK; 3 Division of Epidemiology and Biostatistics, Department of Global Health, Faculty of Medicine and Health Sciences, Stellenbosch University, Stellenbosch 7602, South Africa; 4 Department of Global Public Health, Karolinska Institutet, Tomtebodavägen 18A, Widerströmska Huset, 171 77 Stockholm, Sweden

**Keywords:** neonatal mortality, education, sub-Saharan Africa

## Abstract

Background: Sub-Saharan African (SSA) newborns are ten times more likely to die in the first month than a neonate born in a high-income country. The objective of this study was to examine the relationship between educational attainment and neonatal mortality (NM) among women with cesarean section (CS) deliveries in SSA countries. Methods: Using data from recent demographic and health surveys from 33 countries in SSA, we applied propensity score matching to estimate the effect of education attainment on post-CS neonatal mortality using a propensity-matched cohort where being educated was defined as completing at least primary school education Results: The number of reported CS births ranged from 186 in Niger to 1695 in Kenya. The odds of neonatal mortality between uneducated and educated women ranged from as low as 2.31 in Senegal to 35.5 in Zimbabwe, with a pooled overall risk for NM from all of the countries of OR 2.54 (95% CI: 1.72–3.74) and aOR 1.7 (95% CI: 1.12–2.57). From the 17,220 respondents, we successfully matched 11,162 educated respondents with 2146 uneducated respondents. Uneducated women had a 6% risk compared to a 2.9% risk among educated women for neonatal mortality, with an overall risk of 3.4%; babies from uneducated women were twice as likely to die compared to babies from educated women, RR 2.1 (95% CI, 1.69–2.52). Conclusion: Neonates from uneducated women were twice as likely to die following CS delivery than neonates from educated women. This evidence suggests that a means of achieving Sustainable Development Goal target 3.2 to lower newborn and child mortality is ensuring that everyone has access to high-quality care with efforts made at ensuring education for all and improving socio-economic conditions.

## 1. Introduction

In 2019, SSA had the highest neonatal mortality rate at 27 deaths per 1000 live births; a SSA newborn was ten times more likely to die in the first month than a neonate born in a high-income country [1]. Between 1991 and 2019, global neonatal deaths declined from a median estimate of about 5 million to 2.4 million deaths, with a global neonatal mortality rate (NMR) of 17.5 per 1000 live births. Despite this progress, this decline has not been as significant as seen in under-five deaths (number of deaths in children by age 5 per 1000 livebirths), and neonates consistently made up about 47% of all global under-five deaths between 2017 and 2020 [2].

Frequently, maternal education is seen as one of the most critical determinants of child health outcomes. Extensive research has established a correlation between a mother’s education and her child’s illness and survivability. However, not all findings are clear, and there is little consensus regarding the relationship between a mother’s education and her child’s health [3,4,5,6,7]. The elimination of these discrepancies may present possibilities to save more children’s lives. Ecological studies have shown that sub-optimal national cesarean section (CS) rates (less than 10%) have been associated with high neonatal mortality rates, and this relationship can be mediated or influenced by social determinants of health [8,9,10]. Lower educational attainment is associated with high neonatal mortality rates, and lower educational attainment can be seen along with poor access to adequate information, low awareness of pregnancy complications, delays in decision making on seeking care, delays in seeking care, and inadequate access to quality services. The literature has sought to answer questions concerning the factors associated with CS and NM risk in low- and middle-income countries, including national-level phenomena that explain differences among such countries.

The main purpose of this study was to examine if educational attainment influenced NMR among women with CS deliveries in SSA countries using a propensity-matched cohort.

## 2. Methods

### 2.1. Study Design and Data

This was a secondary data analysis of recent cross-sectional demographic and health survey (DHS) datasets from 33 low- and middle-income countries (LMIC). The DHS surveys are nationally representative household surveys conducted in LMIC, and constitute the largest worldwide effort used for obtaining health and demographic data from developing countries, with reliable quality assurance mechanisms and rigorous survey methods. In this study, we used data from 33 recent DHS surveys conducted between 2010 and 2021 in SSA available as of April 2022. A three-stage stratified cluster sampling with households as the sampling unit is used in these DHS surveys [11]. Within each sample household, all women and men meeting the eligibility criteria are interviewed. These women aged 15–49 years, and men, aged 15–59 years, were interviewed using both men’s and women’s questionnaires. The survey questionnaires are standardized and used across all countries involved in these surveys, with some modifications to suit each country’s needs. The surveys are not self-weighting. Hence, sampling weights are calculated to account for unequal selection probabilities and for non-responses. With these weights, survey findings represent target populations. Data are available for households, including for women, men, and children within these households. These surveys are conducted every five years by ICF International in collaboration with respective national institutions of the countries. In addition, financial support is provided by the US Agency for International Development (USAID). The methods and details of data collection procedures have previously been published [11].

Ethical approval for the survey was granted by the respective countries’ national research ethics committees or equivalent bodies. Informed consent was obtained and participation was voluntary. Access to de-identified datasets was granted by Measure DHS, ICF International USA.

### 2.2. Samples/Dataset

We included cesarean delivery data from the following 33 countries in SSA: Angola, Benin, Burkina Faso, Burundi, Cameroon, Chad, Comoros, Congo, Congo DR, Cote d’Ivoire, Ethiopia, Gabon, Gambia, Ghana, Guinea, Kenya, Lesotho, Liberia, Malawi, Mali, Mozambique, Namibia, Niger, Nigeria, Rwanda, Senegal, Sierra Leone, South Africa, Tanzania, Togo, Uganda, Zambia, and Zimbabwe.

### 2.3. Variables

The outcome variable was neonatal mortality, defined as death occurring less than or equal to 28 days of life and coded as 1 and otherwise 0. The exposure variable was maternal education, categorized as no formal education (1) or educated (0), whereby the respondent had at least completed primary education. Primary education is generally the first phase of formal learning for school-age children, the duration of which is variable in different countries but six years on the average (i.e., primary one to six). 

### 2.4. Covariates

Covariates included the age of the mother in completed years (≤24, 25–34, and 35–49), the mother’s place of residence as rural or urban, wealth index quintiles (poorest, poorer, middle, richer, and richest), the number of antenatal care visits during pregnancy from none, one to four visits, and five or more visits, and media access (a dichotomized variable representing any form of access to the media or no access (magazine, television, or radio)). The wealth grouping in the DHS is a proxy measure of the long-term standard of living based on ownership of certain goods and social facilities by individual households [12]. Briefly, each household had an index of economic status constructed using a principal components analysis based on several household variables: the number of rooms per house, or ownership of car, motorcycle, bicycle, fridge, television, and telephone including any kind of heating device [ibid].

### 2.5. Statistical Analysis

The unit of analysis was any delivery within the last five years preceding the survey. Our analytical approach included descriptive statistics, a meta-analysis, and a propensity score matching analysis. Using basic descriptive statistics, we summarized variable proportions as absolute numbers and percentages, mean, and standard deviations, as applicable. Country weights were used for descriptive statistics in this study. We examined and calculated NM rates at country-level before describing and analyzing women who received CS. 

### 2.6. Individual Patient Data (IPD) Meta-Analysis

For each country, we generated odds ratios (OR) for the relationship between educational attainment and neonatal mortality. To calculate pooled OR across nations, we utilized the DerSimonian–Laird technique (random-effects model) [13]. Cochran’s Q test was used to assess the results’ homogeneity. The metric *I*^2^ denotes percentage variation among heterogeneous investigations [14]. Negative *I*^2^ values were adjusted to zero (no heterogeneity) to produce an *I*^2^ range of 0 to 100%, with greater values indicating increasing heterogeneity [15,16].

### 2.7. Propensity Score Matching Analysis

Since babies born to uneducated women were more likely to be at a higher risk of neonatal mortality due to individual and contextual factors, we used propensity score matching to ensure that the uneducated and educated groups in this study were comparable in terms of important covariates. A standardized difference of 10% or more was suggestive of imbalance. The propensity score approach was used to minimize potential biases in factors that might influence assignment and outcome. To construct this balanced sample, birth-specific propensity scores were estimated from a logistic regression model, which included covariates examined in this study. To create matches and evaluate the quality of matching, simple nearest-neighbor matching with one neighbor (and no replacement) was used. After matching, we examined the quality of matching and gauged comparability of the matched groups using a graph inclusive stata command called ‘pstest’. Having obtained a balanced matched sample, we conducted a pre- and post-match descriptive analysis comparing between-group differences in baseline characteristics for births between uneducated vs. educated women. Finally, we estimated the effect (average treatment effect ATE) of uneducated women on NM outcomes among CS-born babies using ATE, which measures the impact of no-education and whether CS will result in NM. We calculated the absolute difference in the probability of NM among uneducated and educated women in the propensity score-matched cohort. All analyses were completed using Stata version 14 for Windows (StataCorp, College Station, TX, USA). The null hypothesis was tested against a two-sided alternative hypothesis and the statistical significance level was set at *p* < 0.05.

## 3. Results

### 3.1. Summary of Respondents’ Characteristics

**Figure 1:**
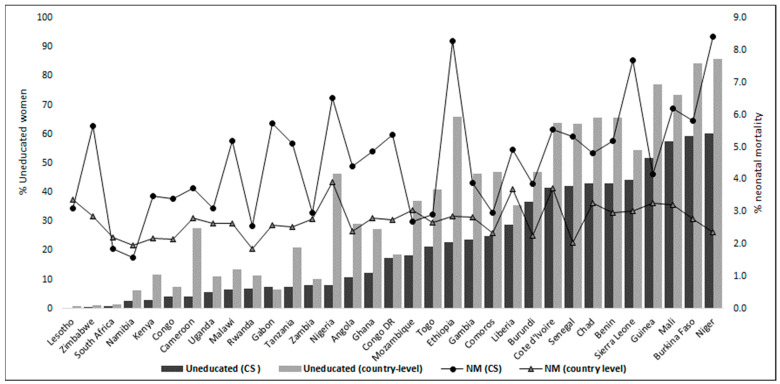
Proportion of uneducated women and neonatal mortality at country level compared to those in the caesarean section population.

From a combined population of 358,529 births, a total of 17,220 respondents (weighted count 18,266) from 33 countries with CS births were included in this study. The number of reported CS births ranged from 186 in Niger to 1695 in Kenya. Three-quarters of the women were aged 25 years and above and belonged to the middle to richest wealth quintiles. While about four-fifths of the women had access to a form of media, the majority (~98%) had at least one antenatal visit during pregnancy. The proportion of neonatal deaths and distribution of educational attainment is shown in Figure 1. Of all of these CS births, 84.1% (15,356/18,262) of respondents had some form of education while 15.9% (*n* = 2907/18,262) had none. The following West African countries constituted the countries with the highest population of uneducated women in this study’s CS population: Niger (60.1%), Burkina-Faso (59.2%), Mali (57.4%), Guinea (51.8%), Sierra-Leone (44.1%), Benin (43.1%), Chad (43%), Senegal (42.1%), and Cote d’Ivoire (41.6%). Southern and East African countries had more educated women. NM rates were generally higher in women who received CS compared to their country-level estimates.

The characteristics of unmatched or matched respondents are summarized in Table 1. There were significant differences between uneducated and educated respondents before matching in wealth, residence, maternal age, media access, and antenatal care visits. More uneducated women resided in rural areas compared to urban areas: 62.1% vs. 43.2% *p* < 0.001. The women were older in the uneducated vs. educated group (35.3% vs. 24.1%). More educated women were richer than uneducated women. About half of all of the respondents had more than four antenatal care visits, whereas less than 2% did not (ANC data availability for respondents was 77%). Between both groups, having more than four ANC visits was observed in 36.5% vs. 51.5% of cases, *p* < 0.001, uneducated vs. educated, respectively. More than 80% of them had at least one form of access to media; about a third of the uneducated women and less than a fifth of the educated women did not have access to any form of media.

### 3.2. Crude Differences in Neonatal Mortality Risk across All Countries between Uneducated Versus Educated Women

**Figure 2:**
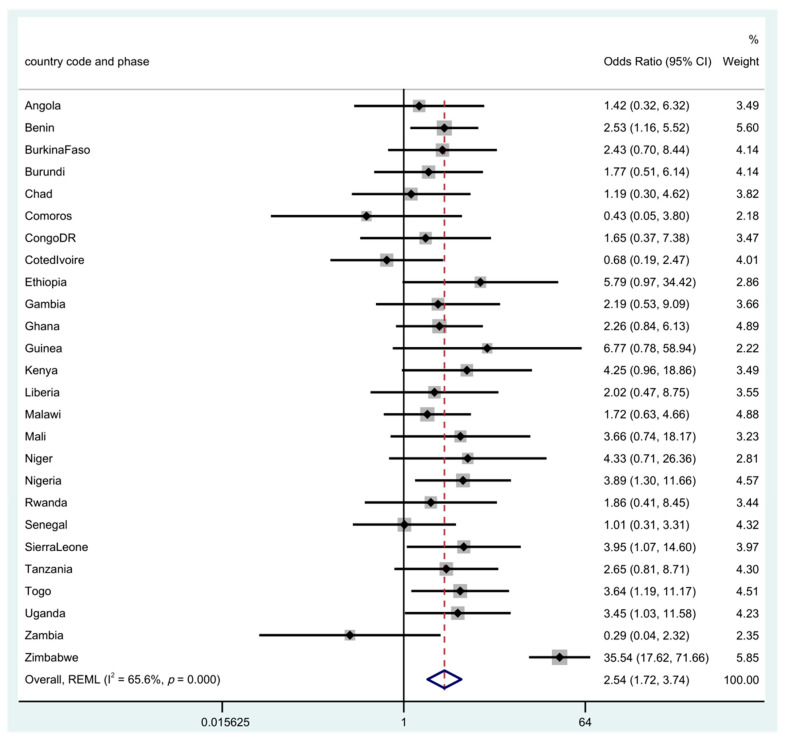
Odds ratio for neonatal mortality in the uneducated compared to educated women. There were 7 studies with missing or insufficient data (Cameroon, Congo, Gabon, Lesotho, Mozambique, Namibia, South Africa). **Note:** Rhomb in blue is the overall ‘pooled’ result of the meta-analysis using restricted maximum likelihood (REML).

**Figure 3:**
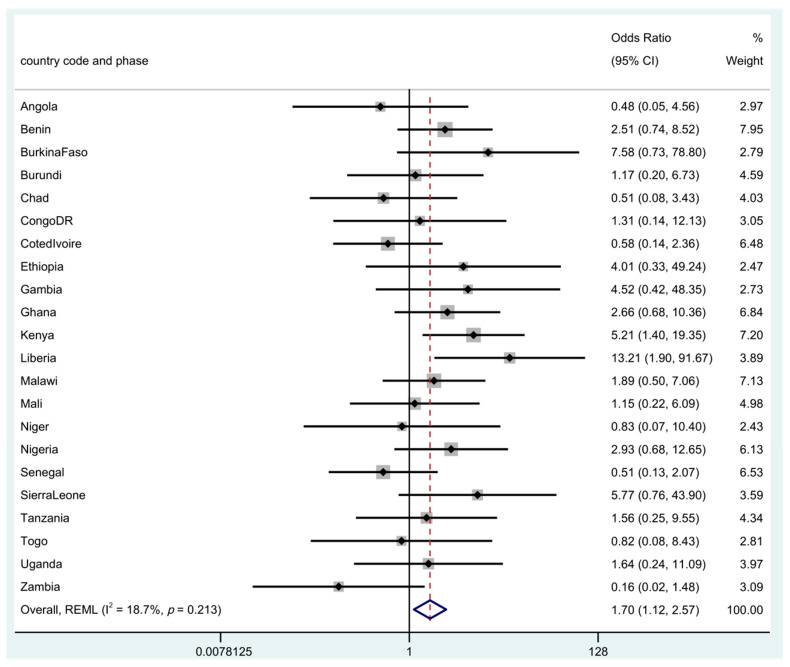
Adjusted odds ratio for neonatal mortality in the uneducated compared to educated women. There were 11 countries with missing or insufficient data: (Cameroon, Comoros, Congo, Gabon, Guinea, Lesotho, Mozambique, Namibia, Rwanda, South Africa, Zimbabwe). **Note:** Rhomb in blue is the overall ‘pooled’ result of the meta-analysis using restricted maximum likelihood (REML).

The odds of neonatal mortality risk between uneducated and educated women are shown in Figure 2 and Figure 3. In the unadjusted individual patient meta-analysis (IPD), and in an increasing order, a significant risk in neonatal mortality from newborns of uneducated women was seen in, Benin, Uganda, Togo, Nigeria, Sierra Leone and Zimbabwe. This ranged from as low as 2.53 in Benin to 35.5 in Zimbabwe with a pooled overall risk for NM from all of the countries of OR 2.54 (95% CI: 1.72–3.74). In the adjusted analysis, neonatal mortality risks in the countries were attenuated and observed in only two countries: Kenya (aOR 5.21; 95% CI, 1.40–19.35) and Liberia (aOR 13.2; 95% CI, 1.9–91.67). The overall pooled estimate from the meta-analysis shows that there is a strong association between maternal education and NM with a risk of aOR 1.7 (95% CI, 1.12–2.57).

### 3.3. Propensity Score-Adjusted Uneducated–Educated Differences in Neonatal Mortality Risk Prevalence

From the 17,220 respondents, we successfully matched 11,162 educated respondents with 2146 uneducated respondents. Uneducated women had a 6% risk compared to a 2.9% risk in educated women for neonatal mortality, with an overall risk of 3.4%; babies from uneducated women were twice more likely to die compared to babies of educated women RR 2.1 (95% CI, 1.69–2.52).

## 4. Discussion

In this study, we examined the effect of educational attainment and NM in CS births in SSA countries using a propensity-matched cohort. We observed that newborns of uneducated women were twice more likely to die compared to newborns of educated women, despite CS intervention.

Overall, most women with cesarean deliveries in the SSA countries were aged 25 to 49 years, were middle class or rich, lived in urban areas, and had access to media and antenatal care. There was, however, a wide geographic variation in the educational attainment of these women. More women from western SSA were uneducated. In the unmatched cohort, uneducated women were older, poorer, and lived in rural areas with a lower number of ANC visits and access to any form of media.

### 4.1. Education Related Inequality in Neonatal Mortality Risk Prevalence

While overall neonatal mortality rates ranged from 19 to 39 per 1000 live births in each country, it was as high as 84 per 1000 live births in CS births, where about 14 countries had rates of 50 and over per 1000 live births. When we examined education alone in the meta-analysis, Nigeria, Togo, Uganda, and Zimbabwe—where more than three-quarters of women who received CS were educated—had higher risks for NM than Benin, which had less than three-fifths of women educated. Kenya, with about 97% of educated women, had a five times greater likelihood of NM when we adjusted for other factors. The risk in Liberia also increased after adjustment for other factors. In the matched cohort, the risk for NM doubled for uneducated women.

Although education is not causally related to neonatal mortality, it is a social determinant of health. Social determinants of health (SDH) are non-medical factors that influence health outcomes. These include the conditions in which people are born, grow, work, live, and age, as well as people’s access to power, money, and resources [17]. In our study, there was a wide variation in the educational attainment of women. These inter-country differences are probably due to differences in country characteristics, structures, and policies addressing social determinants.

Population level studies have shown that where access to CS was higher, neonatal mortality was lower, including among uneducated women. Several studies have reported that low levels of education increased the chances of neonatal death. In Fonseca and McKinnon’s studies [18,19], educational inequality remained in NMR even after adjustment for other factors. According to Kiross [20], when compared with infants of illiterate women, there was a 45% and 28% reduction in infant mortality among secondary or higher educated women and primary educated women, respectively. Similarly, Ratnasiri et al. [21] showed that women who had less than a high school education were 89% more likely to experience infant deaths when compared with women with a bachelor’s degree or higher. In a study of neonatal mortality in Nordic countries, the lowest educated women had a 19–72% higher risk compared with the highest educated women [22].

Being educated increases a mother’s knowledge about pregnancy, the benefits of antenatal care, use of media, health information use, informed birth decisions, healthcare seeking behavior, and awareness about and signs of a sick newborn. Education also influences a woman’s socioeconomic status and autonomy. Findings from surveys in LMIC have shown that a mother’s education contributes to the variation in infant and childhood mortality. In a more in-depth exploration and a slightly contrary opinion. However, a meta-analysis by Mensch et al. [23] showed that the effect of women’s educational outcomes on health outcomes is not as strong as others have argued. This is in line with those who posit that the social pattern differs between neonatal and post-neonatal death, and that post-neonatal mortality has repeatedly been reported to be more closely associated with socioeconomic status than neonatal mortality [22]. This is based on the supposition that when compared with deaths in a later period, socioeconomic factors have less influence on neonatal mortality. Instead, high quality obstetric health care and the provision of social welfare services override social inequalities. The authors of [14] did, however, acknowledge that differences in quality of care may not explain the differences in neonatal mortality observed in their study population. In LMIC however, timely access to and quality of care is still a major problem where CS may be too little or too late compounded by perinatal complications and poor outcomes [24,25]. Thus, except in a few countries, we observed that NM rates were higher in women who received CS relative to country-level estimates.

### 4.2. Strengths and Limitations

The use of a propensity score adjustment method created quasi-randomization, making the adjusted estimates closer to effects seen in a randomized experiment. Although this technique should reduce the bias created by non-random assignment, we acknowledge that there could be residual confounding, given that we do not have other factors to adjust for and cannot be sure that our covariate adjustments were fully adequate. The number of CS deliveries in some countries were low giving rise to insufficient data, missing countries or wide confidence intervals for example as seen in Figure 1 and Figure 2 with particularly wide confidence interval in Zimbabwe. In addition, however, quality of care was not a covariate that could be considered whereas the quality of health care received could influence NM after CS more than the educational status attained by the mother. Another important limitation of the study is bias that could be introduced by period effect. The surveys were conducted over a period of a decade, due to different timing of surveys across countries and comparing two or more groups over the study period.

Given that many health systems are weak in many SSA countries, it is important that these uneducated women are seen as a vulnerable group adequately factored into maternal and childcare planning and policies. Along with other government institutions, education should be a basic human right for all.

## 5. Conclusions

Neonates from uneducated women were as twice as likely to die after CS delivery than neonates from educated women. This evidence suggests that a means of achieving Sustainable Development Goal target 3.2 to lower newborn and child mortality is ensuring that everyone has access to high-quality health care irrespective of their educational status while also ensuring women’s educational attainment is improved by governments.

## Figures and Tables

**Table 1:** Baseline characteristics of uneducated and educated before and after propensity score matching.

	Before Matching				After Matching			
	Uneducated	Educated			Uneducated	Educated		
Variable	%	%	*p*-Value	% Bias	%	%	*p*-Value	% Bias
Educational attainment								
Urban	37.9%	56.8%			37.9%	39.2%		
Rural	62.1%	43.2%	<0.001	38.6	62.1%	60.8%	0.397	2.6
Respondent’s current age (years)								
15–24	20.1%	27.7%			20.1%	21.0%		
25–34	44.6%	48.2%	<0.001	−7.2	44.6%	46.1%	0.327	−3
35–49	35.3%	24.1%	<0.001	24.6	35.3%	32.9%	0.101	5.2
Wealth index combined								
Poorest	22.5%	9.2%			22.5%	25.2%		
Poorer	19.1%	12.5%	<0.001	18	19.1%	17.1%	0.088	5.5
Middle	19.7%	15.1%	<0.001	12.1	19.7%	20.0%	0.76	−1
Richer	21.6%	22.1%	0.649	−1.1	21.6%	20.9%	0.576	1.7
Richest	17.2%	41.1%	<0.001	−54.4	17.2%	16.8%	0.745	0.9
No. of ANC visits during pregnancy								
None	3.6%	1.4%			3.6%	3.3%		
1 to 4	59.9%	47.1%	<0.001	25.9	59.9%	60.4%	0.708	−1.1
5 or more	36.5%	51.5%	<0.001	−30.5	36.5%	36.3%	0.874	0.5
Media Access								
None	35.1%	14.7%			35.1%	36.6%		
Access	64.9%	85.3%	<0.001	−48.6	64.9%	63.4%	0.294	3.7

## Data Availability

The data supporting this article are available at: http://dhsprogram.com/data/available-datasets.cfm (accessed on 25 April 2022).
